# Delayed Umbilical Extrusion of a Ventriculoperitoneal Shunt Following Robotic-Assisted Laparoscopic Hysterectomy: A Case Report

**DOI:** 10.7759/cureus.116324

**Published:** 2026-09-15

**Authors:** Alejandro M Castañer, Amanda Detres, Alejandro E Cedeño-Morán, Miguel J Álvarez-Cortés, Christian Ríos-Vicil, Alexandra Ortiz Oramas

**Affiliations:** 1 Obstetrics and Gynecology, Centro Medico Episcopal San Lucas, Ponce, PRI; 2 Neurosurgery, University of Puerto Rico, Medical Sciences Campus, San Juan, PRI

**Keywords:** distal catheter migration, minimally invasive surgery, umbilical extrusion, ventriculoperitoneal shunt, vp shunt migration

## Abstract

Ventriculoperitoneal shunts (VPSs) are the standard treatment for hydrocephalus but are associated with significant long-term complications, including distal catheter migration. Umbilical extrusion is an exceptionally rare manifestation, particularly in adults, and has seldom been described following minimally invasive gynecologic surgery.

We report the case of a 35-year-old female patient with a VPS placed in early childhood who presented with persistent serous drainage from a prior umbilical port site four months after a robotic-assisted laparoscopic hysterectomy. Initial imaging demonstrated an intra-abdominal distal catheter without extrusion. Over time, the patient developed an extrusion of the catheter through the umbilicus. Abdominal CT confirmed distal migration through the anterior abdominal wall, while head CT showed no hydrocephalus. The patient remained neurologically intact. Surgical management included staged removal of the distal catheter and subsequent removal of the remaining components due to local infection, resulting in complete clinical recovery.

Umbilical extrusion of a VPS catheter is among the rarest reported complications, with most cases occurring in pediatric populations. In this case, prior laparoscopic surgery may have contributed by altering intra-abdominal pressure dynamics, disrupting tissue planes, and weakening fascial integrity at port sites, thereby facilitating catheter extrusion. The prolonged latency highlights the lifelong risk of VPS-related complications and suggests that previously stable catheters may become susceptible to extrusion following abdominal interventions. Persistent postoperative umbilical drainage in patients with VPS should prompt evaluation for catheter extrusion. Awareness of this rare complication is essential for surgeons performing laparoscopic procedures in patients with indwelling shunts. Multidisciplinary management and appropriate perioperative planning are critical to optimize outcomes.

## Introduction

Ventriculoperitoneal shunting is a commonly used procedure for managing hydrocephalus, diverting cerebrospinal fluid (CSF) from the cerebral ventricles to the peritoneal cavity [1]. Despite its effectiveness, shunt malfunction may necessitate revision [1,2]. Reported complications include obstruction, infection, shunt fractures, pseudocysts, ascites, and distal catheter migration [1-4]. Umbilical extrusion is among the rarest forms of distal migration. A systematic review of the literature from 1973 to 2021 identified 27 reported cases and included 24 of these cases in its detailed analysis, all involving children [5]. Proposed mechanisms include chronic irritation, silicone allergy, and foreign body reaction, although the underlying pathophysiology remains uncertain [4]. Distal catheter migration following abdominopelvic surgery has also been reported, including migration through the vaginal cuff after laparoscopic hysterectomy [6]. Distal catheter migration is classified as internal, external, or compound; umbilical extrusion represents external migration, in which the catheter penetrates the body wall [7]. We present the case of a 35-year-old woman with a ventriculoperitoneal shunt (VPS) placed in early childhood who developed persistent umbilical drainage approximately four months after robotic-assisted laparoscopic hysterectomy and cystoscopy for chronic pelvic pain and abnormal uterine bleeding, followed by confirmed catheter extrusion approximately 10 months after surgery. To our knowledge, umbilical extrusion of a VPS catheter in an adult after robotic-assisted gynecologic surgery has rarely been reported.

This case was previously presented as a meeting poster at the 21st Caribe Gyn Meeting on August 7, 2025, in Ponce, Puerto Rico.

## Case presentation

A 35-year-old G3P2012 female patient presented with a history of VPS placement in 1992 for hydrocephalus secondary to a benign intracranial dermoid tumor, which was resected at that time. After VPS placement, the patient was lost to neurosurgical follow-up throughout childhood, adolescence, and adulthood. Past medical history was significant for endometriosis, diagnosed after an exploratory laparotomy in 2016 for chronic pelvic pain and menorrhagia. Other surgical interventions included cardiac valve repair at six months of age, cesarean delivery in 2013, and repeat cesarean delivery in 2020.

In August 2024, she underwent a robotic-assisted laparoscopic hysterectomy for chronic pelvic pain attributed to endometriosis. The distal VPS catheter was visualized intraoperatively but not manipulated. The patient was discharged home on postoperative day 1 without complications. Four months postoperatively, persistent clear, serous drainage was noted from the umbilical port site. A computed tomography (CT) scan was obtained to evaluate for a postoperative seroma or fluid collection. No periumbilical fluid collection or evidence of catheter extrusion was identified, and the distal catheter tip remained intra-abdominal (Figure 1). The patient was managed conservatively with wound care and outpatient follow-up. She continued to experience intermittent serous drainage from the umbilical site without associated systemic symptoms. In June 2025, extrusion of the distal VPS catheter through the umbilicus was identified during a wound care visit, with a small segment protruding through the central umbilical defect, surrounded by mild periumbilical erythema and localized skin irritation (Figure 2). She was referred to the Emergency Department, where repeat abdominal CT confirmed distal catheter migration (Figure 3A), while brain CT demonstrated a stable proximal catheter without hydrocephalus (Figure 3B). The patient remained neurologically intact, afebrile, and without signs of systemic infection.

**Figure 1:**
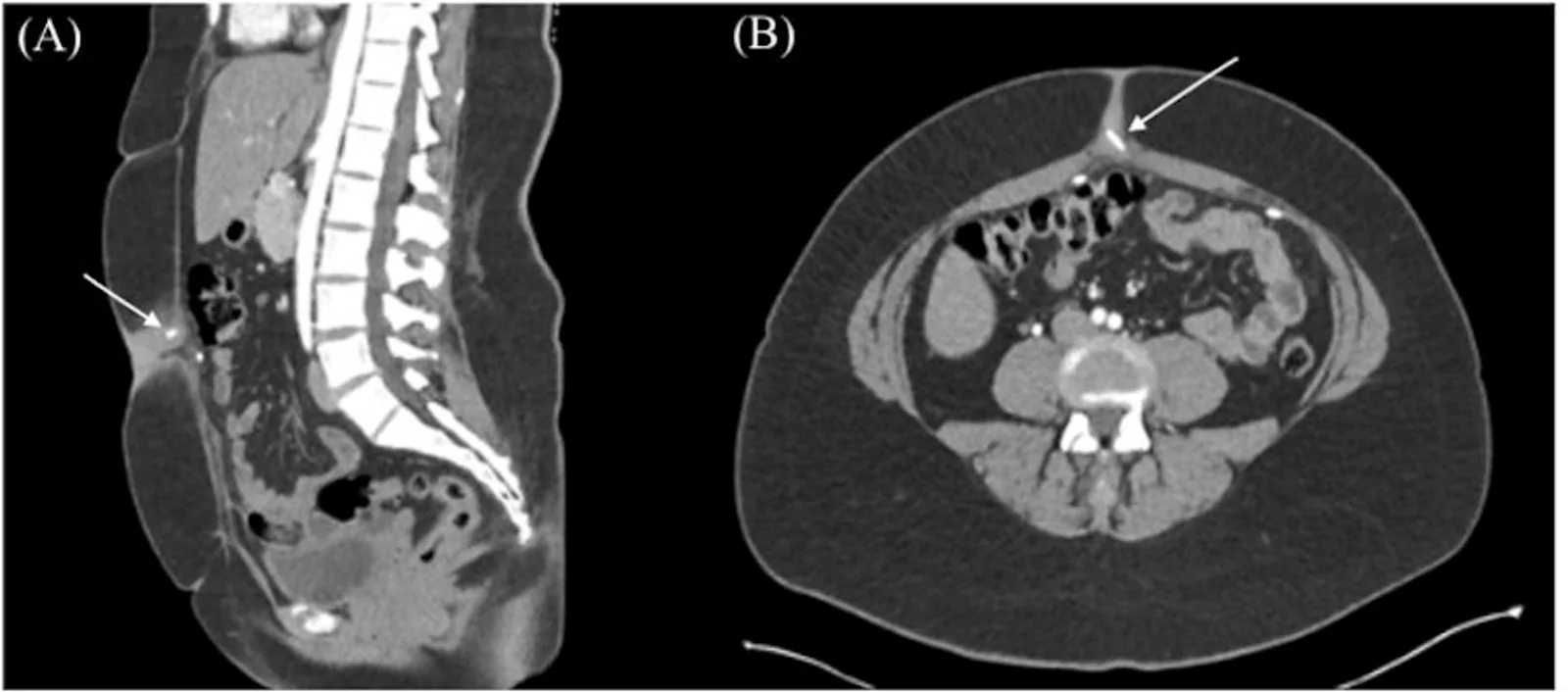
CT of the abdomen and pelvis. Sagittal (A) and axial (B) views demonstrate the distal ventriculoperitoneal shunt catheter positioned posterior to but not protruding through the umbilicus. CT, computed tomography

**Figure 2:**
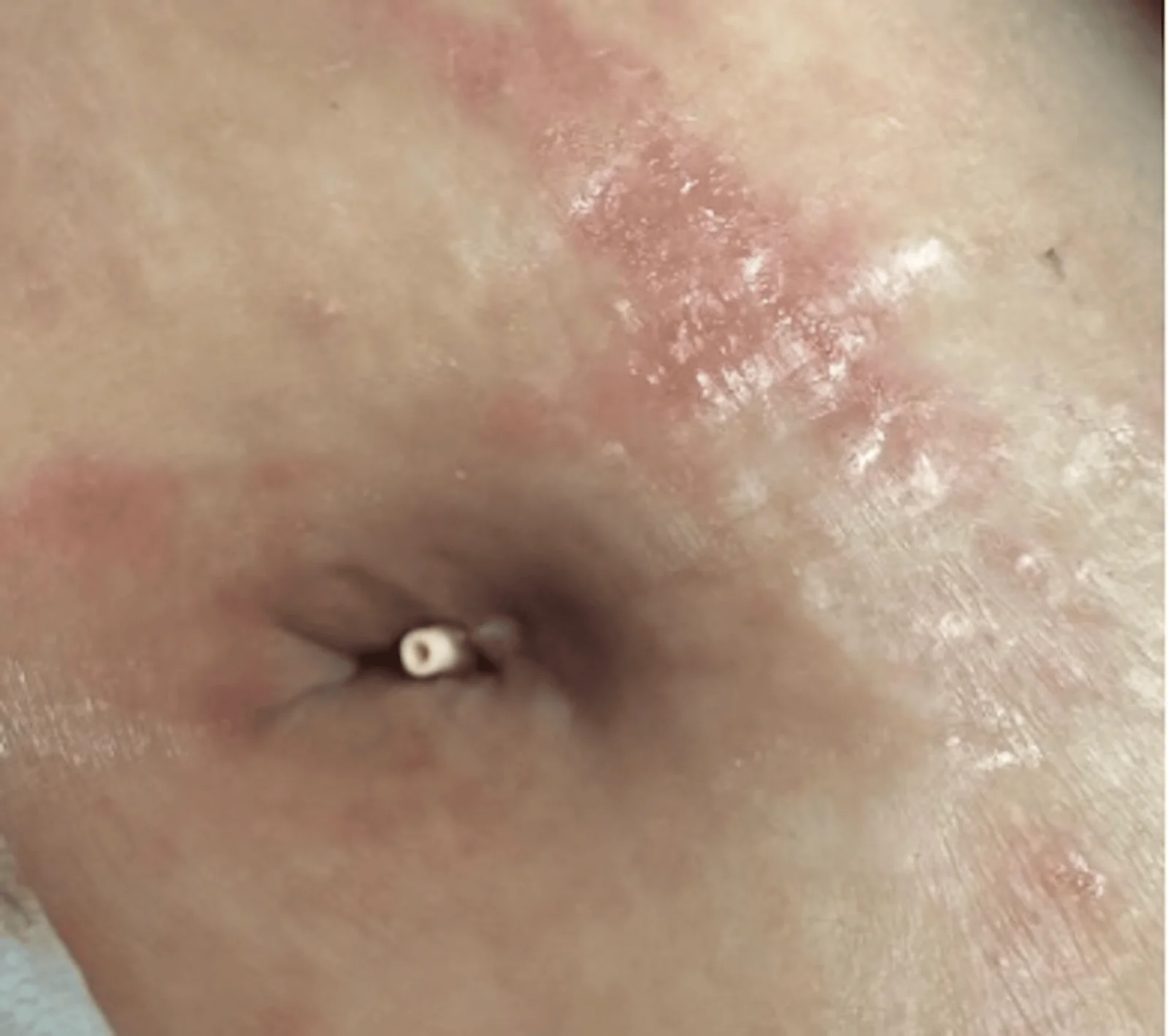
Clinical photograph of the abdomen demonstrating extrusion of the distal ventriculoperitoneal shunt catheter through the umbilicus, surrounded by mild periumbilical erythema and localized skin irritation, without gross purulence or active bleeding.

**Figure 3:**
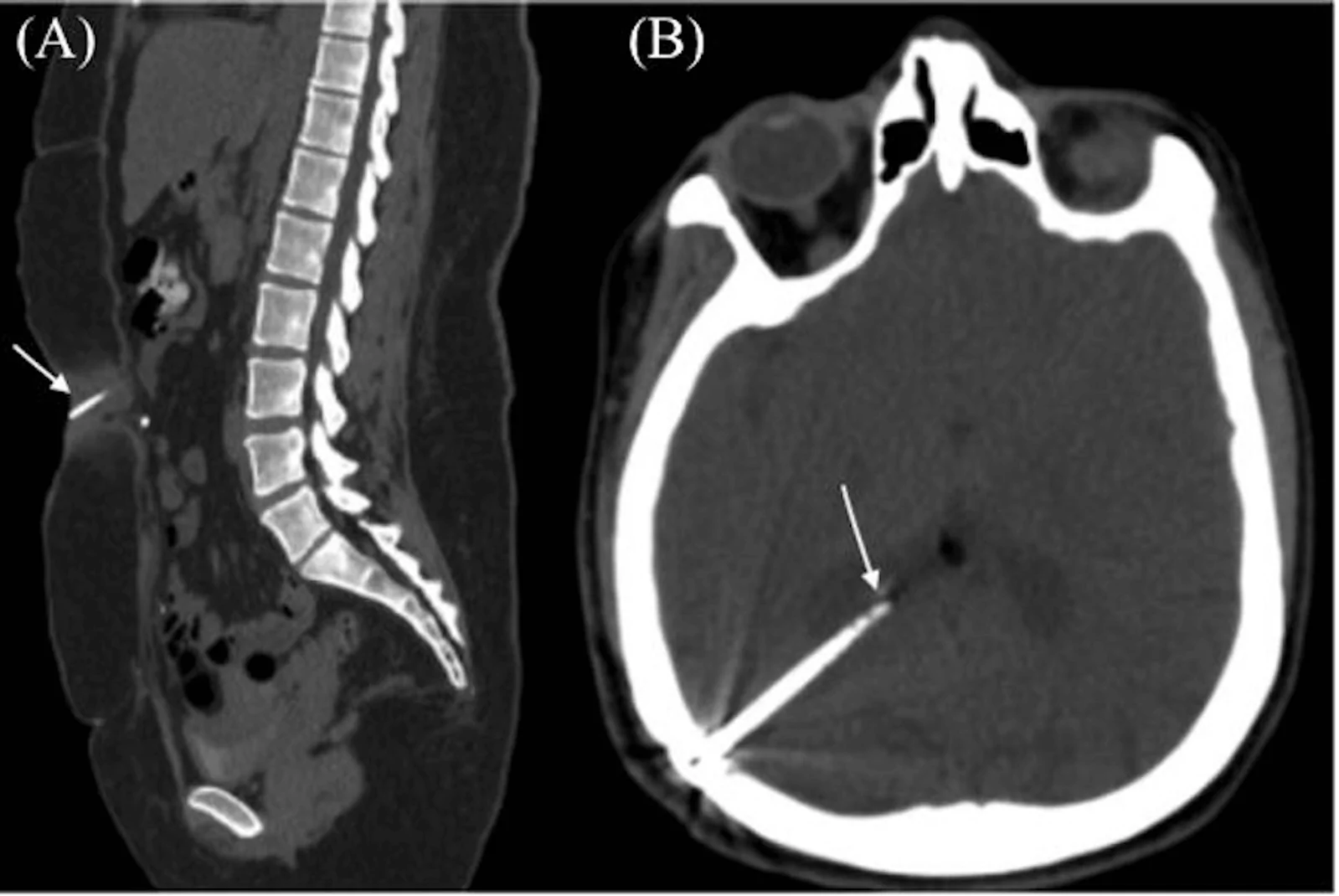
Radiologic evaluation of VPS position. (A) CT of the abdomen and pelvis (sagittal view) demonstrates the distal VPS catheter with migration toward the anterior abdominal wall and umbilical region. The white arrow indicates the distal catheter. (B) Non-contrast CT of the head (axial view) shows the proximal VPS catheter without ventricular enlargement or evidence of hydrocephalus. The white arrow indicates the proximal catheter tip within the ventricular system. CT, computed tomography; VPS, ventriculoperitoneal shunt

On June 30, 2025, the neurosurgery team performed partial removal of the distal catheter at the level of the clavicle. The proximal catheter and valve system were left in place due to dense fibrosis and proximity to the choroid plexus, with concern for potential intracranial hemorrhage if removed. Intraoperatively, the shunt was determined to be nonfunctional due to lack of spontaneous CSF egress. During the same operation, general surgery was consulted intraoperatively. Exploration of the umbilicus revealed granulation tissue and a small umbilical hernia measuring less than 1 cm. The granulation tissue was removed, and the area was cauterized. The hernia was repaired primarily using a figure-of-eight 0 Vicryl suture, and the umbilical skin was closed with interrupted 3-0 Monocryl sutures. No complications were reported. The patient remained clinically stable and asymptomatic, suggesting the shunt had likely been nonfunctional prior to extrusion.

On July 16, 2025, the patient was readmitted with a localized infection at the catheter stump near the right clavicle. Laboratory studies revealed a white blood cell count of 10.53 Thou/mL, reactive thrombocytosis (platelet count, 497 Thou/mL), a slightly elevated C-reactive protein level of 6.6 mg/L, and a normal erythrocyte sedimentation rate of 7 mm/h. Empiric intravenous therapy with cefepime 1 g and vancomycin 1 g was initiated. The infectious diseases team recommended increasing the cefepime dose to 2 g to ensure adequate central nervous system penetration given the possibility of intracranial involvement, and adding metronidazole 500 mg for anaerobic coverage. A superficial wound culture from the right cervical surgical site grew *Klebsiella pneumoniae*. The isolate was susceptible to amoxicillin-clavulanate, amikacin, ceftazidime, ceftriaxone, cefepime, gentamicin, meropenem, imipenem, and trimethoprim-sulfamethoxazole; intermediate to ampicillin-sulbactam and levofloxacin; susceptible-dose dependent to piperacillin-tazobactam; and resistant to cefoxitin. On July 25, 2025, the patient underwent reoperation to remove the remaining valve and distal catheter remnant. Cefepime provided definitive coverage, and a 14-day course of intravenous antibiotic therapy was completed on August 1, 2025. She remained neurologically stable without recurrence of symptoms.

## Discussion

We report a rare case of delayed umbilical extrusion of a VPS distal catheter in a 35-year-old woman, occurring more than 30 years after initial shunt placement for childhood hydrocephalus and almost one year after a robotic-assisted laparoscopic hysterectomy. The patient underwent successful surgical management with removal of the distal catheter (and valve), primary umbilical hernia repair, and targeted antibiotic therapy, with no neurological sequelae.

Abdominal complications of ventriculoperitoneal shunts

Although VPS remains the most commonly performed surgical intervention for hydrocephalus, it carries a significant long-term complication burden. Mechanical failure occurs in more than 50% of children within two years of placement, with a lifetime revision risk of at least 80% [8]. Abdominal complications related to the distal catheter are reported in 10-30% of cases and represent the most frequent category of VP shunt failure [8]. These include shunt obstruction, infection, CSF pseudocyst formation, bowel obstruction, and catheter migration or coiling [9]. Reported extrusion sites include the rectum/anus, oral cavity, scrotum, vagina, urinary bladder, umbilicus, and, in exceptionally rare instances, the lumbar back or paraspinal musculature [8,10]. Bowel perforation carries an associated mortality of up to 15%, with only approximately 25% of affected patients presenting with the classic features of peritonitis [11]. Among the various extrusion sites, umbilical extrusion is exceptionally uncommon and will be the focus of this discussion.

Umbilical ventriculoperitoneal shunt extrusion

Umbilical CSF fistula and extrusion of the distal VPS catheter through the umbilicus are among the rarest reported complications. Since the first report by Adoleye et al. in 1973, only a limited number of cases have been described in the literature [12-18]. Most reported cases have occurred in infants and children, often in association with congenital umbilical defects, prior central nervous system infections, abdominal adhesions, or other predisposing anatomical abnormalities [3,19-20]. Recent pediatric series have identified umbilical migration as an uncommon manifestation of distal catheter displacement, with only isolated cases reported among larger cohorts of shunt migration complications [8-10]. Adult presentations remain exceedingly rare. In a systematic review by Ghritlaharey, the median interval from shunt insertion to diagnosis was four months, and the longest documented latency was eight years [5]. Our patient developed extrusion more than three decades after VPS placement, which may represent one of the longest latency intervals reported in the literature. The temporal association with prior laparoscopic surgery raises the possibility that altered intra-abdominal pressure dynamics, disruption of tissue planes, and weakened fascial integrity at port sites contributed to extrusion, although a direct causal relationship cannot be established.

Pathophysiology

The exact mechanism underlying umbilical extrusion remains poorly understood, and multiple pathophysiological pathways have been proposed. The most widely cited mechanism involves mechanical pressure exerted by the distal catheter tip against adjacent abdominal wall structures, resulting in local inflammation, sterile peritonitis, and progressive fibrosis around the shunt tube [3,20]. This fibrotic reaction anchors the catheter to surrounding visceral or parietal surfaces; CSF pulsations then generate a repetitive water-hammering effect that gradually erodes tissue planes until a fistulous tract is established [8].

The umbilicus represents the weakest point of the anterior abdominal wall and is therefore a natural site of convergence for migrating catheters [20]. In infants and children, a patent vitello-intestinal duct or urachal remnant may provide a preformed channel for catheter migration [3,19]. Mohindra et al. proposed five possible mechanisms: anatomical weakness, umbilical abscess, persistent umbilical vein, perivisceritis, and persistent urachal remnant, later adding an abnormally patent intercoelomic communication [18]. Early presentations (within six months of surgery) are often associated with surgical site or shunt infection, whereas delayed presentations are more commonly attributable to congenital anatomical anomalies or gradual mechanical erosion [19]. In adult patients, prior abdominal surgery, chronic fibrosis, and progressive tissue erosion from a long-indwelling catheter are the most plausible contributing factors in the absence of congenital anomalies. Shunt material properties also play a role: silicone allergy, firm catheter tips, and biological reactivity have all been identified as contributing factors to distal migration [8].

In our case, several mechanisms may plausibly explain the temporal association between robotic-assisted hysterectomy and delayed catheter extrusion. Although insufflation has not been shown to increase intracranial pressure or precipitate valve failure in patients with shunts [6], transient pneumoperitoneum may still alter the position of a chronically adherent distal catheter, and trocar placement and postoperative fibrosis may disrupt established tissue planes. Furthermore, surgical manipulation and adhesion remodeling may create lower-resistance pathways that facilitate progressive distal migration over time.

Clinical presentations

The clinical presentation is variable but frequently subtle. In most reported cases, the first sign is a clear or yellowish fluid discharge from the umbilicus, which, when positive for beta-transferrin, confirms a CSF fistula [3]. In the series by Ezzat et al., although mostly in pediatric patients, the most common presenting symptom across all migration types was vomiting (66.7%), followed by bulging of the anterior fontanelle and visible extrusion of the catheter from the migration site [8]. In cases of umbilical extrusion specifically, the distal catheter tip may not be visible on initial examination [3]. Vankipuram et al. described a pouting granulation tissue at the umbilical opening with purulent discharge, suggesting secondary infection overlying the underlying fistula tract [19].

A clinically silent course was observed in our case. Imaging obtained months before definitive extrusion showed no evidence of localized fluid collection or catheter extrusion. Abdominal radiography and CT imaging are useful for confirming the distal catheter tip position and identifying any associated fluid collection [3,20]. Brain CT or MRI should also be obtained to assess ventricular size [19]. In pediatric series, the mean interval from initial shunt placement to the diagnosis of a complication ranges from approximately 7 to 13 months, though our case illustrates that presentation can be delayed for decades, particularly when abdominal surgery disrupts a previously stable catheter-tissue interface. Persistent postoperative umbilical drainage in a patient with a VPS should be considered a potential warning sign of impending catheter migration, even when initial imaging does not demonstrate extrusion.

Management

Management of umbilical VPS extrusion must be individualized based on the extent of catheter extrusion, evidence of infection, shunt functionality, and residual intraperitoneal catheter length [10]. The standard approach consists of removal of the exposed distal catheter, external CSF diversion if the proximal shunt is functional, intravenous broad-spectrum antibiotics, and delayed shunt reinsertion after exclusion or resolution of peritoneal infection [8,11]. Shunt salvage has been reported in selected cases. Pant et al. demonstrated successful salvage in 3 of 17 patients with distal migration, including one case of umbilical extrusion in which only 3 cm of the catheter had traversed beyond the umbilicus. The authors emphasized that the adequacy of residual intraperitoneal shunt length must be confirmed before considering salvage, as insufficient length may not accommodate somatic growth in pediatric patients [10]. Similarly, Xia et al. described a laparoscopic approach in which only the extruded portion of the catheter was resected, and the remaining intra-abdominal end was repositioned on the diaphragmatic surface of the liver, thereby preserving shunt function and reducing operative costs and risks [20]. When the shunt is nonfunctional, complete removal of exposed components, along with wound management and antibiotic therapy, is appropriate [19]. Antibiotic therapy should be guided by CSF cultures and administered for at least three weeks; shunt replacement should follow confirmed sterilization [11].

## Conclusions

Umbilical extrusion of a VPS catheter is an extremely rare but serious delayed complication that may occur decades after initial placement. Reported cases have almost exclusively involved young children with short intervals between shunt insertion and extrusion, making the present case, occurring in an adult more than three decades after placement and shortly after robotic-assisted laparoscopic hysterectomy, an unusual presentation. Awareness of this complication is essential for obstetricians and gynecologists who perform pelvic laparoscopic procedures. Careful preoperative imaging, neurosurgical consultation, and port placement are critical in patients with pre-existing VPS. Successful management requires a multidisciplinary approach encompassing neurosurgery, general surgery, and infectious disease, with individualized decisions regarding shunt salvage, removal, or replacement guided by catheter functionality, infection status, and neurological condition. This case underscores the importance of maintaining clinical suspicion for VPS complications in patients presenting with persistent postoperative wound drainage and highlights the need for long-term monitoring of patients with indwelling shunt systems.
